# Outcome of 313 Czech Patients With IgA Nephropathy After Renal Transplantation

**DOI:** 10.3389/fimmu.2021.726215

**Published:** 2021-09-30

**Authors:** Dita Maixnerova, Petra Hruba, Michaela Neprasova, Kamila Bednarova, Janka Slatinska, Miloslav Suchanek, Marek Kollar, Jan Novak, Vladimir Tesar, Ondrej Viklicky

**Affiliations:** ^1^ Department of Nephrology, General Teaching Hospital, 1st Faculty of Medicine, Prague, Czech Republic; ^2^ Department of Nephrology, Institute of Clinical and Experimental Medicine, Prague, Czech Republic; ^3^ University of Jan Evangelista Purkyně in Ústí nad Labem, Ústí nad Labem, Czech Republic; ^4^ Department of Pathology, Institute of Clinical and Experimental Medicine, Prague, Czech Republic; ^5^ Department of Microbiology, University of Alabama at Birmingham, Birmingham, AL, United States

**Keywords:** IgA nephropathy, kidney transplantation, the recurrence of IgA nephropathy, microscopic hematuria, proteinuria, renal failure

## Abstract

The recurrence of IgA nephropathy (IgAN) after kidney transplantation occurs in 20–35% of patients. The main aim of this study is to evaluate risk factors affecting the course of IgAN after renal biopsy of native kidney and kidney transplant. We evaluated clinical parameters and histological findings at the time of biopsy of native kidney and after kidney transplantation in 313 patients with IgAN with a follow-up of up to 36 years. Using hierarchical clustering method, patients with graft failure (n=50) were divided into two groups based on the mean time from kidney transplant to graft failure (11.2 *versus* 6.1 years). The time-to-graft failure corresponded well to the time from the renal biopsy of native kidney to end-stage renal disease (5.9 *versus* 0.4 years). Body mass index, proteinuria, microscopic hematuria, histological evaluation of fibrosis, and crescents at the time of renal biopsy of native kidney were the main variables for the differentiation of the two groups. Higher age of kidney-transplant donor, histological recurrence of IgAN, antibody-mediated rejection, and the onset of microscopic hematuria and proteinuria within 1 year after kidney transplant were also associated with worse graft survival in multivariate Cox regression analysis.

## Introduction

IgA nephropathy (IgAN) is the most common primary glomerulonephritis with potentially serious outcome leading to end-stage renal disease in 30% to 50% of patients within 20 to 30 years from diagnosis. The diagnosis of IgAN requires evaluation of a renal biopsy specimen and finding of IgA immunodeposits (1). A multihit pathophysiological process has been proposed for the development of IgAN, starting with the production of galactose-deficient IgA1 and the corresponding autotantibodies driving the production of pathogenic immune complexes, with genetic and environmental contributing factors (2, 3). Clinical risk factors predicting poor prognosis include time-averaged proteinuria, hypertension, decreased estimated glomerular filtration rate (eGFR) (2, 4), as well as histological lesions characterized by the Oxford classification (MEST-C score) (1, 5, 6). The decision for additional treatment options beyond the recommended RAS blockade should respect the chance of inducing clinical remission, reducing proteinuria, hematuria, stabilizing renal function without further decline of GFR, as well as mitigating the risk of adverse events that may occur with, for example, using corticosteroids and other types of immunosuppression. Thus, the consideration of the risks and benefits needs to be made in individual cases. An early initiation of immunosuppressive regimen in patients with IgAN at risk of progression may slow down the progression to end-stage renal disease. Several new treatment options for patients with IgAN are currently under evaluation in clinical trials (7).

Due to the young age and minimal comorbidities, patients with IgAN are optimal candidates for kidney transplantation. However, the recurrence of the original disease in the graft is a known complication in patients with IgAN and an often cause in the decline of graft function. Other glomerular or vascular lesions secondary to acute or chronic graft rejections might also negatively affect the graft function.

To better understand the graft function, we evaluated in 313 patients with IgAN who received kidney transplant clinical parameters and histological findings at the time of biopsy of native kidney performed in 1991–2013 and after receiving the kidney transplant in 1996–2017. The main aim of our study is to assess the outcome of renal transplants in this cohort. We identified risk factors for disease progression after kidney transplantation and assessed the correlation with the previous rate of decline of renal function in the native kidneys.

## Materials and Methods

Our study is a retrospective, multicenter, observational study of patients with biopsy-confirmed IgAN who received kidney transplant at the Institute for Clinical and Experimental Medicine, Prague, Czech Republic. We retrieved all patients with biopsy-confirmed IgAN from Czech registry of renal biopsies (n=2,377) performed in 25 hospitals in the Czech Republic between 1991 and 2017, and all patients transplanted between 1996 and 2017 at the Institute for Clinical and Experimental Medicine (n=523) and their overlap resulted in a final cohort of 313 patients.

Clinical parameters (erythrocyturia, proteinuria, serum creatinine, eGFR, blood pressure, BMI) were retrieved at the time of native kidney biopsy. In addition, crescents and fibrosis were evaluated in biopsy with native IgAN. After kidney transplantation, all transplant parameters (including peak PRA, HLA mismatch, cold ischemia time, dialysis vintage, recipient and donor ages, and genders) were extracted from the hospital database. The occurrence of rising proteinuria (>0.5 g/24 h) and erythrocyturia (>10 erythrocytes/1 µl of urine sediment) and all kidney-biopsy findings were also included in analyses. Kidney-biopsy findings for each case were evaluated independently by two specialized nephropathologists. Any unclear cases were discussed with an additional nephropathologist to reach a consensus.

Native kidney biopsies were performed in local hospitals indicated due to impaired renal function and/or urinalysis and evaluated centrally by pathologists from the Institute for Clinical and Experimental Medicine. After transplantation, besides indication biopsies, the center standard-of-care 3-month protocol biopsies were performed for all kidney-transplant recipients. Indication biopsies were performed based on observed deterioration of graft function and/or proteinuria. The decision for renal biopsy after kidney transplantation was made by the medical team of at least six nephrologists. Only 8% (25/313) patients never had allograft biopsy.

The evaluation of patients with performed renal biopsies was approved by the Ethics Committee of the General University Hospital in Prague, Czech Republic (number 1443/11, S-IV). Written informed consent was obtained from all subjects involved in the study who underwent renal biopsy.

### Statistical Analyses

Agglomerative hierarchical clustering was used for the differentiation of primary data (time from renal biopsy to dialysis, from renal biopsy to transplantation, or from transplantation to graft failure) into two groups. Discriminant analysis with transformed (principal) variables was used to predict the membership of the other variables to the group. The verification of membership was confirmed by Mann–Whitney nonparametric test. Logistic regression was used for the validation of the results of the discriminant analysis and for the calculation of ROC curves in the case of two groups. Response type for logistic regression was binary; prediction accuracy was nearly 100%. All calculations were done by the program XLSTAT (www.xlstat.com), Continuous variables were compared by the Mann–Whitney U test and categorical variables by Pearson’s chi-squared test. The death-censored graft survival was evaluated by Kaplan–Meier analysis using the log-rank test. To investigate the significance of each prognostic factor for allograft loss, univariable Cox proportional hazards models were calculated for clinical parameters at the time of native kidney biopsy (age and gender, proteinuria, erythrocyturia, creatinine), and clinical variables related to kidney transplantation (HLA mismatch, cold ischemia, dialysis vintage, donor age and gender, type of donor, repeated transplantation, proteinuria and erythrocyturia, biopsy-proven reIgAn, and ABMR) were assessed in a univariable model. Potential predictors of graft loss were included in the multivariable model based on their significance in the univariable analysis. P values of <0.05 were considered significant. Statistical analysis was performed using IBM SPSS Statistics, Version 24 (International Business Machines Corp.) and GraphPad Prism 5, Version 5.03 (GraphPad Software, Inc.).

## Results

**Figure 1:**
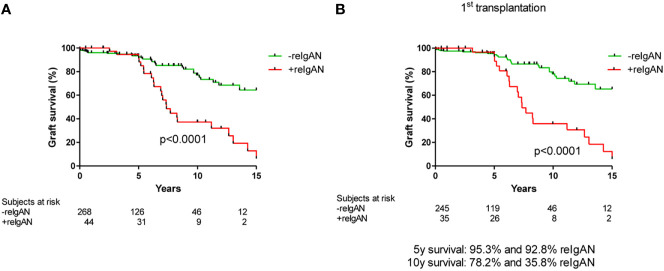
Death-censored graft survival in patients with/without histological recurrence of IgAN in **(A)** total cohort (log rank p value p < 0.0001) and **(B)** only in recipients who received their first graft (log rank p value p < 0.0001).

**Figure 2:**
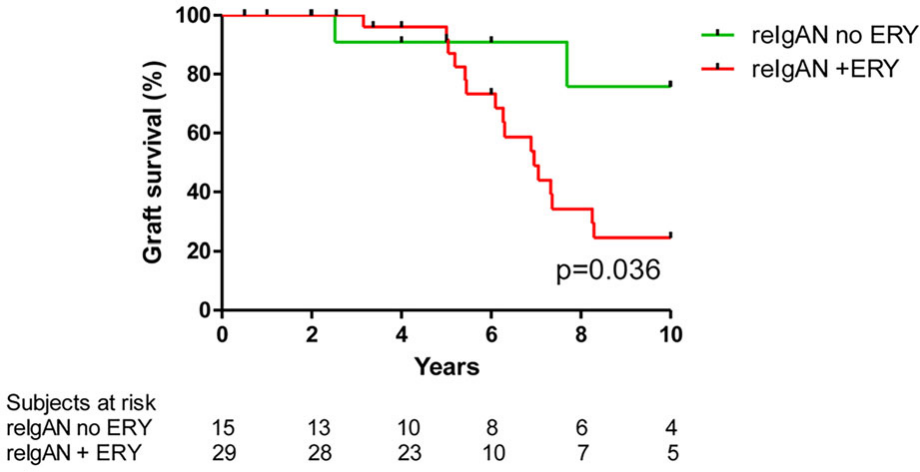
Death-censored graft survival in patients with histological recurrence of IgAN with/without microscopic hematuria. Patients with both reIgAN and microscopic hematuria had worse graft survival (log rank p value=0.036). ERY, microscopic hematuria; reIgAN, recurrence of IgAN.

We evaluated 313 patients with histological diagnosis of IgAN in native kidney biopsies who later received kidney transplants. Detailed characteristics of the cohort are in **Tables 1** and **2**. Forty-four of these patients (14%) exhibited histological recurrence of IgAN in the graft. Except for younger age of patients with IgAN recurrence, no other predictive factors for recurrence of IgAN were identified [recipient gender, donor age, donor gender, retransplantation, living donor, peak PRA (panel reactive antibody), HLA mismatch, dialysis vintage (length of time on dialysis), cold ischemia; **Table 2**]. Ten-year renal survival was substantially reduced in patients with histologically diagnosed recurrence of IgAN compared to patients without recurrent IgAN (**Figure 1**). In 23 patients with recurrence of IgAN, the grafts failed (52.3%). The main causes of graft failure were recurrence of IgAN (n= 17), recurrence of IgAN in combination with ABMR (n=2), with TCMR (n=3), and a mixed rejection (n=1). In patients without histological recurrence, 35 grafts failed (13%). The main causes of graft loss were rejections (ABMR, n=11; TCMR, n=2; mixed rejection, n=2), infections (n=6), surgery complication in early post-transplant period (n=5), chronic allograft nephropathy (n=4), and cardiorenal syndrome (n=2). Erythrocyturia was detected in 29 (65.9%) of the 44 patients with recurrent IgAN. Thus, erythrocyturia (>10 erythrocytes/1 µl of urine sediment), common in IgAN patients with native kidneys, was not observed in 34% of patients with recurrent IgAN. Ten-year renal survival was unfavorable in patients with histologically confirmed recurrent IgAN and microscopic hematuria compared to those with recurrent IgAN but without microscopic hematuria (**Figure 2**).

**Table 1:** The evaluation of 313 Czech patients with histological diagnosis of IgAN (30 patients with two kidney transplantations, 3 patients with three kidney transplantations).

Patients	Numbers	Period from RB to dialysis (years)^a^	Period from RB to Tx (years)^a^	S-Cr levelat diagnosis(µmol/L)^a^	Proteinuriaat diagnosis(g/day)^a^
After 1. Tx	280	2.99	4.91	246	2.05
Graft failure after 1. Tx	50	1.33	2.42	301	1.15
Preserved renal function after 1. Tx	230	3.41	5.82	230	2.35

^a^Median value

IgAN, IgA nephropathy; Tx, kidney transplantation; RB, renal biopsy; S-Cr level, level of serum creatinine.

**Table 2:** Patients with histological recurrence of IgAN, significant young recipient’s age.

	Total cohort	Histological recurrence of IgAN		
	**n = 313**	**yes (n = 44)**	**no (n = 269)**	**p value**
Recipient age, years [range]	46 [20,79]	38 [20,62]	47 [22,79]	<0.0001
Recipient gender, male, n (%)	253 (81%)	35 (80%)	218 (81%)	0.837
Donor age, years [range]	53 [6,81]	53 [21,73]	53 [6,81]	0.588
Donor gender, male, n (%)	135 (43%)	14 (49%)	121 (36%)	0.167
Retransplantation, n (%)	33 (10.5%)	9 (21%)	24 (9%)	0.032
Living donor, n (%)	90 (29%)	14 (32%)	76 (28%)	0.72
Peak PRA [range]	4 [0,96]	4 [0,96]	4 [0,92]	0.757
HLA mismatch [range]	3 [0,6]	3 [2,6]	3 [0,6]	0.856
Dialysis vintage, months [range]	16 [0,97]	16 [0,78]	16 [0,97]	0.694
Cold ischemia, hours [range]	13 [0,26]	13 [0,23]	13 [0,26]	0.882
Erythrocyturia post Tx	74 (24%)	29 (65.9%)	45 (17.3%)	<0.001
Proteinuria post Tx	90 (29%)	33 (75%)	57 (21.2%)	<0.001
Follow-up, years [range]	5 [0, 21]	6 [0.5, 15]	4 [0, 21]	0.881
Graft failure n (%)	58 (18.5%)	23 (52.3%)	35 (13%)	<0.001
Time to graft failure, years [range]	5 [6.3,17.5]	6.9 [2.5,15.4]	5.3 [0.03,17.5]	0.074

**Figure 3:**
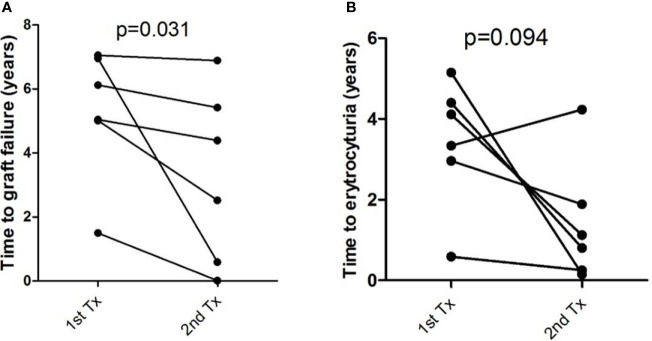
Time to **(A)** graft failure and **(B)** erythrocyturia in six patients with repeated transplantation. Patients after second transplantation had shorter time to graft failure (p = 0.031) and nonsignificantly shorter time to erythrocyturia (p = 0.094). Differences were calculated by Wilcoxon signed rank test.

Of the total of 313 patients, 33 underwent repeat kidney transplantation (**Table 1**). Graft failure developed in six patients after second kidney transplantation; these patients had shorter time to the appearance of erythrocyturia both after the first and second renal transplantations (**Figure 3**).

**Figure 4:**
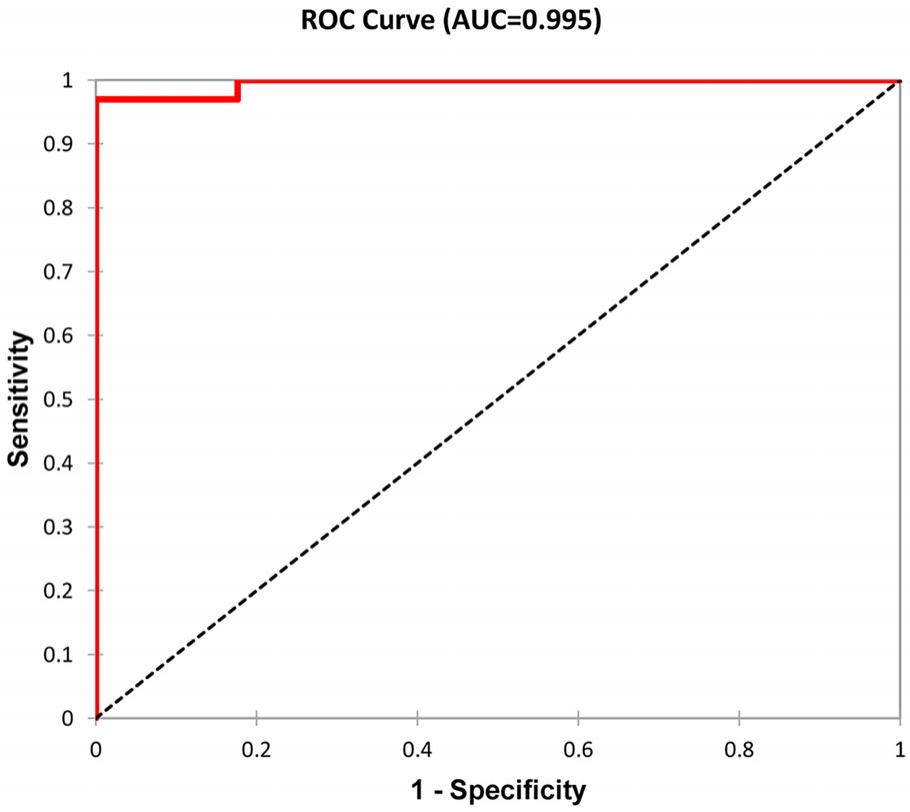
ROC curve as a graphical representation of logistic regression. Patients were distributed according to the time from kidney transplant to graft failure and the time from renal biopsy to end-stage renal disease into two groups (see **Table 3**). A total of 50 patients with graft failure were divided into two groups with 17 and 33 members. Classification accuracy was 96%.

Subsequently, we evaluated 50 patients (16%) with graft failure after the first kidney transplant. Erythrocyturia occurred in 31 (62%) of these patients. Histologically verified recurrence of IgAN was confirmed in 23 individuals (46%). According to the hierarchical clustering method, 50 patients were divided into two groups that were compared using Mann–Whitney test (**Table 3**). Statistical parameters of both groups are shown in **Table 4**. Logistic regression analysis confirmed the distribution, as shown in an ROC curve (**Figure 4**). The time from kidney transplant to graft failure (10.0 *versus* 5.5 years) correlated well with the distribution of the two groups based on the time from renal biopsy of native kidney to end-stage renal disease (6.8 *versus* 1.1 years).

**Table 3:** Two groups of patients with original diagnosis of IgAN after kidney transplant and graft failure were differentiated by agglomerative hierarchical clustering.

Statistical parameters						
Statistic	From RB to dialysis (years)		From RB to Tx (years)		From Tx to graft failure (years)	
	group 1	group 2	group 1	group 2	group 1	group 2
Nbr. of obs.	17	33	17	33	17	33
Median	5.9	0.4	7.1	2.2	11.2	6.1
Mean	6.8	1.1	6.8	2.3	10.0	5.5
SD	4.8	1.4	4.5	1.4	4.5	3.2
Mann-Whitney test	p < 0.0001		p = 0.001		p = 0.001	

RB, renal biopsy; Tx, kidney transplant.

**Table 4:** Age, gender, renal parameters, proteinuria, erythrocyturia, fibrosis, crescents, age of transplantation, blood pressure, BMI, weight, and height at the time of renal biopsy in two groups of patients with original diagnosis of IgAN after kidney transplant with graft failure.

	Slow progressors (group 1)	Rapid progressors (group 2)	p value
N	17	33	
Age at RB, years	33.5 (9.9)	34.9 (12.3)	0.49
Patient gender, male, n (%)	11 (65%)	27 (82%)	7.8*
Creatinine at RB (μmol/L)	330 (184)	399 (277)	0.633
eGFR CKD EPI (mL/s)	0.47 (0.36)	0.48 (0.46)	0.739
PU at RB (g/24 hours)	1.52 (1.72)	2.93 (2.88)	0.077
Erythrocyturia at RB, n (%)	15 (88%)	23 (74%)^2^	8.1*
Fibrosis at RB, n (%)	11 (79%)^3^	22 (85%)^7^	7.9*
Crescents at RB, n (%)	4 (29%)^3^	17 (59%)^4^	6.1*
Age of transplantation, years	40 (10)	37 (12)	0.325
SPB RB	155 (29)	144 (19)	0.285
DPB RB	99 (22)	86 (18)	0.208
BMI RB	24 (3)	26 (4)	0.033
Weight RB (kg)	73 (10)	78 (19)	0.105
Height RB (cm)	174 (11)	171 (8)	0.632

*Test of proportion; calculated Z value (normal distribution) from transformed binomial distribution; Z (p = 0.05) = 1.96.

Numbers in superscript indicate number of missing values.

eGFR, glomerular filtration rate (ml/s); PU, proteinuria (g/day); SBP, systolic blood pressure; DBP, diastolic blood pressure; RB, renal biopsy of native kidney; BMI, body mass index.

Quantitative parameters: mean (SD); qualitative parameters: group frequency (%).

Females were overrepresented in the group with a slower loss of renal function (female to male ratio 0.54 *versus* 0.22). Other risk factors, such as body mass index, proteinuria, microscopic hematuria, and histological evaluation of fibrosis and crescents at the time of renal biopsy of native kidney, were also confirmed, but additional factors, including age, renal parameters, and blood pressure at the onset of renal biopsy of native kidney, did not affect the differentiation into two groups (**Table 4**).

Higher age of the kidney-transplant donor, histologically verified recurrence of IgAN, antibody-mediated rejection, and the onset of microscopic hematuria and proteinuria within 1 year after kidney transplant were associated with worse graft survival in multivariate Cox regression analysis for patients with original diagnosis of IgAN in native kidney biopsy (**Tables 5** and **6**).

**Table 5:** Analysis of risk factors for graft survival in patients with IgAN in biopsy of native kidney.

	P value	HR	95% CI for HR	
			Lower	Upper
**EU till 1 year after Tx**	0.000	4.638	2.562	8.394
**PU till 1 year after Tx**	0.000	7.236	3.315	15.797
**reIgAN**	0.000	3.305	1.937	5.640
**ABMR**	0.001	3.242	1.653	6.358
**Age—recipient**	0.001	0.961	0.939	0.984
**Age—RB**	0.002	0.962	0.939	0.986
**Retransplantation**	0.060	2.167	0.967	4.855
Recurrence to 2y after Tx	0.106	2.214	0.844	5.806
Gender—donor, male	0.112	0.606	0.326	1.124
Age—donor	0.145	1.018	0.994	1.042
S-Cr at RB	0.189	1.001	1.000	1.002
PRA max	0.220	0.992	0.979	1.005
EU native kidney	0.471	0.777	0.390	1.545
Mismatch	0.551	1.078	0.842	1.381
CIT hours	0.626	1.009	0.973	1.047
Gender—recipient, male	0.686	1.145	0.593	2.212
Dialysis (months)	0.788	1.002	0.986	1.018
PU at RB	0.857	0.990	0.888	1.104
Type of donor deceased	0.975	1.009	0.577	1.764

reIgAN, recurrence of IgAN histologically verified; RB, renal biopsy; ESRD, end-stage renal disease; Tx, kidney transplant; EU, microscopic hematuria; PU, proteinuria; CIT, time of cold ischemia; S-Cr, the level of serum creatinine; ABMR, antibody mediated reaction; 2y, two years.

Cox regression model univariate.

**Table 6:** Multivariate Cox regression model for graft survival.

	Sig.	HR(Exp B)	95.0% CI for Exp (B)	
			Lower	Upper
Donor´s age	**0.043**	1.027	1.001	1.054
Recipient´s age	0.258	0.983	0.954	1.013
reIgAN	**0.023**	2.426	1.129	5.216
ABMR	**0.000**	5.503	2.359	12.837
EU till 1 year after Tx	**0.008**	3.119	1.341	7.251
PU till 1 year after Tx	**0.049**	2.791	1.003	7.767

reIgAN, the recurrence of IgAN histological verified; ABMR, antibody mediated rejection; EU, microscopic hematuria till 1 year after kidney transplant; PU, proteinuria till 1 year after kidney transplant.

Graft survival in patients with original diagnose of IgAN was influenced by histological verified ABMR (HR = 5.5, p < 0.0001), recurrence of IgAN (HR = 2.4, p = 0.023), the onset of erythrocyturia till 1 year after kidney transplant (HR = 3.1, p = 0.008), the onset of proteinuria till 1 year after kidney transplant (HR=2.8, p=0.049), and higher age of donor (HR = 1.03, p = 0.043).

**Figure 5:**
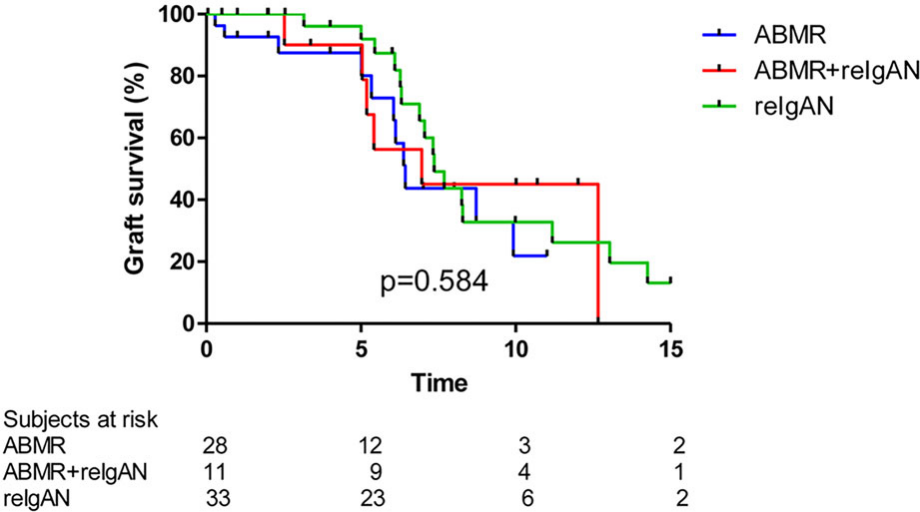
Death-censored graft survival in patients with ABMR, reIgAN + ABMR, and reIgAN is similar (log rank p value p = 0.584). ABMR, antibody mediated rejection.

We did not find differences among subgroups of patients with isolated recurrence of IgAN (i.e., without antibody-mediated rejection), antibody-mediated rejection, or combination of recurrence of IgAN with antibody-mediated rejection on graft survival (**Figure 5**).

## Discussion

Microscopic hematuria is not a reliable manifestation of IgAN recurrence after kidney transplantation. In an Italian cohort of 190 transplanted patients with IgAN, microscopic hematuria was not present at diagnosis of the recurrence in 25% of the patients (8), similar to our results showing absence of microscopic hematuria in 34% of the patients at that time point after kidney transplantation. Another study that assessed early recurrence of IgAN in the graft by protocol biopsy showed that 64% of the patients with recurrent IgAN did not exhibit microscopic hematuria (9). In these patients, histological recurrence of IgAN was found in 1/3 of 65 patients as determined by mesangial IgA immunodeposits in using protocol renal-biopsy specimens with or without mesangial proliferation and with or without mild clinical symptoms (microscopic hematuria and/or proteinuria).

The incidence of IgAN recurrence in biopsies diagnosed based on clinical parameters is approximately 30%, ranging from 9% to 53% among different centers with regard to protocol and clinical biopsies (**Table 7**). This enormous variance might be assigned to diverse biopsy strategies and techniques of histological evaluation in different centers. Thus, the 14% (44 patients) with histologically verified recurrence of IgAN in our cohort of 313 Czech patients falls just inside this range.

**Table 7:** The recurrence of IgAN in kidney transplant.

First author	Year of publication	Number of pts	reIgAN rate	Time to recurrence (years)	Graft losses	FU
Jiang et al. (10)	2018	2393	9.9	4.6		10
Berthoux et al. (11)	2017	96	35.4	5.8	12.3	12.4
Garnier et al. (12)	2018	67	20.8	3.13		5.9
Avasare et al. (13)	2017	62	22.5	2.75		
Von Visger	2014	124	22			6.8
Moroni et al. (8)	2013	190	22.1	3.7	6.3	9.4
Mousson (14)	2007	42	52.4		4.7	8.9
Allen et al. (15)	2017	2501	40.7			7.7

pts, patients; reIgAN, recurrence of IgAN, graft losses due to recurrence of IgAN; FU, follow-up.

The rate of recurrence after kidney transplant might be undervalued in the absence of a histological diagnosis of the original disease in native kidneys or in the case of short follow-up after kidney transplant (16) and/or in patients who did not undergo the graft biopsy with regard to renal-function decline or proteinuria (17). Additionally, it was assumed that the presence of IgA deposits in donated kidneys (from living or deceased donors) might intensify the possibility of recurrence (18, 19). However, when allografts with latent mesangial IgA deposits were transplanted to non-IgAN patients, the IgA immunodeposits were cleared in the recipients, as confirmed by follow-up biopsies (20). Using repeat kidney biopsies, it was shown that in some patients with IgAN, IgA deposits disappear after renal transplantation (21, 22). On the other hand, silent IgA deposits might drive clinical IgAN recurrence and disease progression (23).

The conversion of the histological recurrence into clinical recurrence might be associated with the activation of complement and cytokines production (24). Nevertheless, precise clinical significance of the histological recurrence as well as its effective treatment is unknown; therefore, protocol biopsies in patients with IgAN after kidney transplantation are not recommended. The recurrence of IgAN is a time-dependent event that requires a long follow-up to reach a higher probability of recurrence (8, 10).

In our cohort of 313 patients, 10-year renal survival was unfavorable in patients with histologically recurrent IgAN compared to patients without recurrent IgAN (**Figure 3**). Histologically verified recurrence of IgAN was confirmed in 23 individuals (46%) of the total number of 50 (16%) patients with IgAN with graft failure in our study, which was a higher percentage compared to the literature (25). The rate of graft loss due to recurrence was reported to range from 1.3% (46) to 17% (25). Needless to say, other factors such as glomerular or vascular lesions secondary to acute, chronic graft rejections, or the toxicity of calcineurin inhibitors might influence a graft loss, especially in case of longer time of graft biopsy to the graft function decline (26).

Which predictors of graft failure due to IgAN recurrence in kidney transplant are known? In a cohort of 71 transplanted IgAN patients, cellular or fibrocellular crescents were confirmed in 14% of graft biopsies and were related to higher interstitial inflammation with a significantly worse graft survival (27). The Oxford classification of histological features of IgAN in the native kidney (28) used for biopsies of recurrent IgAN should have prognostic importance for graft failure (29, 30), and, thus, Oxford classification score is suggested to be used for all cases of IgAN recurrence.

The crescents in histological evaluation of native biopsies were an unfavorable factor for renal function of native kidney and for graft survival in our cohort of 313 Czech patients. This finding is in agreement with the results of Columbia University Medical Center from 2001 to 2012 (13). On the other hand, young age at renal transplantation as well as at the onset of IgAN and male gender at renal transplantation reported by others (13, 17, 31, 32) were not confirmed as risk factors for loss of renal function in our study. Other factors related to kidney-transplant survival were not confirmed in our cohort.

We confirmed the expected clinical risk factors predicting poor outcome in patients with native IgAN, such as proteinuria, erythrocyturia, and body mass index (**Table 4**). The relation between the body mass index and the probability of end-stage renal disease was shown in Chinese patients with IgAN (33). An increased body mass index associates with lower remission of proteinuria subsequent to treatment in a Japanese cohort (34). It was assumed that obesity increased proteinuria in connection with hypertension and metabolic syndrome. Moreover, the advantages of losing weight in overweight patients with IgAN with protein and sodium restriction and maximal control of hypertension using treatment with inhibitors of the renin-angiotensin system were confirmed (35).

It was suggested that an increased risk of recurrences and graft loss in patients with IgAN with the first graft loss was due to recurrence (15, 36, 37), which we confirmed in this study. We detected significantly shorter time to erythrocyturia after first and second kidney transplant and shorter time to second kidney transplantation in patients with IgAN (**Figure 3**). On the other hand, 7,236 patients of the ANZDATA transplant registry did not have an increased risk of recurrence with graft loss in a subsequent graft in patients with IgAN (10).

Do living-donor kidneys have a more frequent risk of recurrence and graft failure compared to the kidneys from deceased donors? In our cohort, histologically verified recurrence of IgAN as well as the rate of graft survival in recipients from living-related donors was similar to that of recipients from deceased donors. Some studies showed an unfavorable effect of living donors on the graft outcome (2, 8, 38–41), but other studies failed to confirm this finding (42–44).

We would like to emphasize that our study involves a retrospective analysis of a large cohort of IgAN patients after kidney transplant, comprising data for native kidney biopsies and regular protocolar biopsies of grafts from one center, which, until now, was an underreported area of central Europe regarding this theme. The limitation of our study includes the lack of serum or urinary biomarkers for the assessment of the activity of disease at the time of diagnosis or relapse and/or in association with treatment. Serum IgA levels or serum levels of autoantibodies specific for galactose-deficient IgA1 after kidney transplant or native-kidney biopsy were assessed as the risk factor of IgAN recurrence in other studies (11, 12). Another limitation of our study was due to protocol biopsies that were indicated 3 months after kidney transplantation, and, thus, patients with clinically silent recurrent IgAN would be missed. The use of more frequent protocol biopsies was not possible in our transplantation center. Another limitation of our study is missing information about immunosuppressive therapy, use of ACE inhibitors/angiotensin receptor blockers, or statins. Our survey was a single-center study; most patients received maintenance immunosuppression therapy consisting of calcineurin inhibitors, mycophenolate mofetil, and corticosteroids. Most of our patients receive ACE inhibitors. Therefore, adding this information to the model would not provide any substantial effect.

## Conclusion

Microscopic hematuria with histologically verified recurrence of IgAN predicts an unfavorable renal survival in a cohort of 313 Czech patients with IgAN who received kidney transplant (p < 0.001). Significantly better renal function was found in female graft recipients. We confirmed that higher BMI, male gender, clinical activity (proteinuria, erythrocyturia), and histological findings, including crescents and enlarged interstitial fibrosis, are risk factors for progressive loss of renal function in patients with IgAN. Furthermore, higher age of donor of the kidney predicted worse prognosis of graft survival of patients with IgAN.

## Data Availability Statement

The original contributions presented in the study are included in the article/supplementary material. Further inquiries can be directed to the corresponding author.

## Ethics Statement

The studies involving human participants were reviewed and approved by the ethics committee of 1st Medical Faculty, Charles University, Prague, General Teaching Hospital, Prague. The patients/participants provided their written informed consent to participate in this study.

## Author Contributions

All authors listed have made a substantial, direct, and intellectual contribution to the work and approved it for publication.

## Funding

The authors received funding from grants PROGRES Q25/LF1, DRO VFN 64165, IKEM IN 00023001 from the Ministry of Health of the Czech Republic and NanoEnviCz No. LM2015073 from the Ministry of Education Youth and Sports.

## Conflict of Interest

JN is a co-founder and co-owner of and consultant for Reliant Glycosciences, LLC, and a co-inventor on US patent application 14/318,082 (assigned to UAB Research Foundation).

The remaining authors declare that the research was conducted in the absence of any commercial or financial relationships that could be construed as a potential conflict of interest.

## Publisher’s Note

All claims expressed in this article are solely those of the authors and do not necessarily represent those of their affiliated organizations, or those of the publisher, the editors and the reviewers. Any product that may be evaluated in this article, or claim that may be made by its manufacturer, is not guaranteed or endorsed by the publisher.
